# Representation of nuclear magnetic moments via a Clifford algebra formulation of Bohm’s hidden variables

**DOI:** 10.1038/s41598-022-24970-4

**Published:** 2022-12-18

**Authors:** Ruggero Maria Santilli, Garret Sobczyk

**Affiliations:** 1 Department of Physics, The Institute for Basic Research, 35246 U. S. 19 North, Palm Harbor, FL 34684 USA; 2 Departamento de Acuaria Física y Matemáticas, Universidad de las Américas-Puebla, 72820 Puebla, Mexico

**Keywords:** Nuclear physics, Theoretical nuclear physics, Mathematics and computing, Physics

## Abstract

In this paper, we outline the research conducted by the first named author and his associates on the axiom-preserving, thus isotopic completion of quantum mechanics into hadronic mechanics according to the historical legacy by A. Einstein, B. Podolsky and N. Rosen that *quantum mechanics is not a complete theory* and review the ensuing exact representation of the magnetic moment and spin of the Deuteron in its ground state thanks to the isotopic completion of Pauli’s matrices with an explicit and concrete content of D. Bohm’s *hidden variable* $\lambda$. We then outline the independent studies conducted by the second named author on the representation of the conventional Pauli’s matrices via geometric Clifford algebras. We finally show that the combination of the two studies allows a mathematically rigorous, numerically exact and time invariant geometric representation of the magnetic moment, spin and hidden variable of the Deuteron in its ground state.

## Introduction

In this paper, we combine the studies by the first named author, the physicist R. M. Santilli, and the independent studies by the second named author, the mathematician G. Sobczyk, to present a mathematically rigorous, numerically exact and time invariant representation of the magnetic moment and spin of the Deuteron in its ground state.

We begin with an outline of the inability by quantum mechanics to achieve an exact representation of nuclear magnetic moments under the assumption of the tabulated values of the magnetic moments of the proton and of the neutron in vacuum^1^

1 $$\begin{aligned} \mu _p = + 2.79285 \,\mu _N,\quad \mu _n = - 1.91304\,\mu _N, \end{aligned}$$

where $\mu _N$ is the unit called *nuclear magneton.*

**Figure 1:**
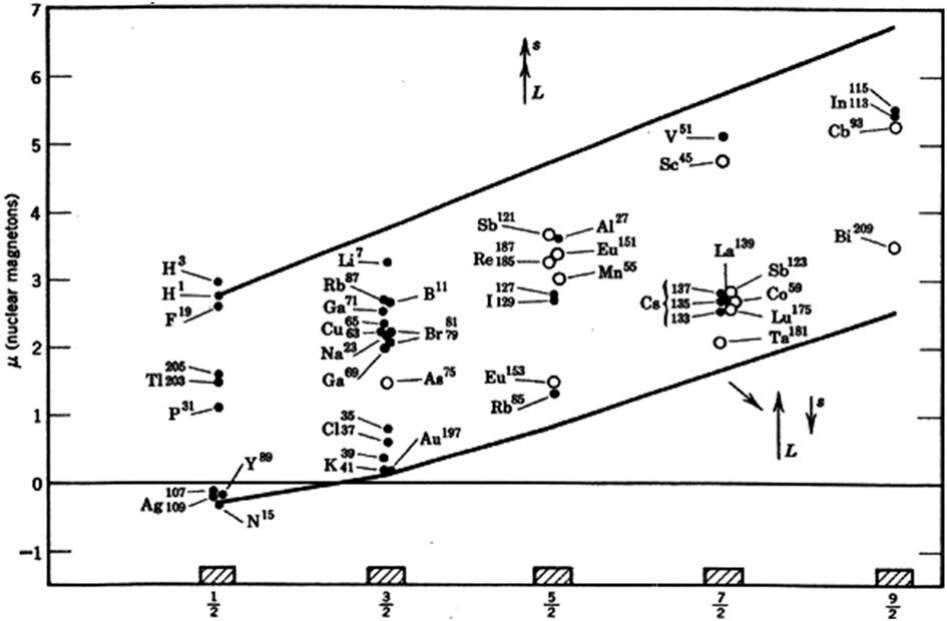
This figure illustrates the well known Schmidt limits in nuclear physics, which establish deviations of the predictions of quantum mechanics for nuclear magnetic moments from their experimental values, beginning with that for the smallest nucleus, the Deuteron. These and other insufficiencies of quantum mechanics in nuclear physics, such as that for nuclear spins, supports the need of a suitable completion of quantum mechanics according to the 1935 historical Einstein–Podolsky–Rosen argument^2^.

As an example, the magnetic moment predicted by quantum mechanics (qm) for the Deuteron under value (1) is given by

2 $$\begin{aligned} \mu _D^{qm} = \mu _p + \mu _n = (2.79285 - 1.91304)\,\mu _N = 0.87981 \mu _N, \end{aligned}$$

while the experimentally measured value is given by

3 $$\begin{aligned} \mu _D^{ex} = 0.85647 \,\mu _N, \end{aligned}$$

resulting in a deviation of the theoretical prediction in *excess* of about $3\%$ of the experimental value,

4 $$\begin{aligned} \mu _D^{qm} - \mu _D^{ex} = 0.02334\,\mu _N \approx 2.95\% \mu _D^{ex}, \end{aligned}$$

with larger deviations for heavier nuclei (Fig. 1).

Additionally, quantum mechanics has been unable to achieve a consistent representation of the spin $S_d = 1$ of the Deuteron *in its ground state,* since the only stable bound state predicted by quantum mechanics at short distance for a proton and a neutron is the singlet with antiparallel spins and null total angular momentum $J_d = 0$. As a result of this occurrence, the spin $S_d = 1$ of the Deuteron is represented via a *collection of orbital contributions* yielding $J_d = 1$^3^ that cannot exist in the ground state; and other unresolved aspects.

Fermi^4^, Weisskopf^3^ and other founders of nuclear physics formulated the hypothesis that *in the transition from isolated particles in vacuum to members of a nuclear structure, protons and neutrons experience a deformation of their extended charge distribution with consequential change of their magnetic moments* (1) *while conserving their spin* 1/2 (see the statement at the top of p. 31 of Refs.^3,5^ for a preliminary experimental verification). Despite its simplicity, the above Fermi-Weisskopf hypothesis has profound mathematical and theoretical implications because it requires the transition from the quantum mechanical representation of particles as non-deformable *points* to a covering representation of particles as *extended,* therefore deformable.

In fact, an important axiomatic limitation of quantum mechanics is precisely its strict *locality,* that is, the sole possibility of characterizing particles as being point-like in vacuum, thus solely admitting action-at-a-distance potential interactions (hereon called *Hamiltonian interactions*).

By contrast, experimental measurements have established that nuclear volumes are generally *smaller* than the sum of the volume of the nucleon constituents. Consequently, nuclei are composed by *extended* nucleons in condition of partial mutual penetration of their dense charge distributions, resulting in Hamiltonian as well as new interactions expected to be non-linear (in the wave function), non-local (because defined over volumes) and not representable with a Hamiltonian (hereon called *non-Hamiltonian interactions*).

Additional experiments have established deviations of the predictions of quantum mechanics from experimental values for: the anomalous magnetic moment of the muons^6^; atoms in condensed matter^7^; heavy ions physics^8^; and other fields. The above insufficiencies of quantum mechanics supports:

1. The historical 1935 argument by A. Einstein, B. Podolsky and N. Rosen that *“quantum mechanics is not a complete theory”* (EPR argument)^2^;
2. The historical completions of quantum mechanics, such as the non-linear completion by Heisenberg^9^, the non-local completion by de Broglie and Bohm^10^, and the completion via *hidden variables* by Bohm^11^;
3. The studies over the past fifty years conducted by R. M. Santilli and other scholars on the EPR completion of quantum mechanics into the axiom-preserving *hadronic mechanics*^12–15^ for the representation of the dimension, shape and density of hadrons with Hamiltonian and non-Hamiltonian interactions (see Ref.^14^ for detailed studies, Refs.^16–20^ for recent accounts, Ref.^21^ for a recent overview, proceedings^22^ on the *2020 International Teleconference on the EPR argument,* and independent works^23–30^).

The above studies have achieved a numerically exact and time invariant representation of the magnetic moment and spin of the Deuteron in its true ground state (that with null orbital contributions $J_d = 0$) thanks to an axiom-preserving, thus isotopic completion of Pauli’s matrices with an explicit and concrete realization of Bohm’s hidden variable $\lambda$, which completion was first introduced in Eq. (6.28.20), p. 354, Vol. II of Ref.^14^, used in various applications such as the verification of the EPR argument^16^, and then applied to the representation of the experimental data of the Deuteron in its ground state, resulting in the numeric value $\lambda = 2.65557$ (see the latest Ref.^31^ and prior references quoted therein).

Independently from the above studies, the mathematician G. Sobczyk has conducted systematic studies on the representation of the conventional Pauli matrices, via geometric Clifford algebras (see Refs.^32–36^ and references quoted therein).

In this paper we show that, by joining the studies by R. M. Santilli and G. Sobczyk, geometric Clifford algebras can provide a mathematically rigorous, numerically exact and time invariant representation of the magnetic moment, spin and hidden variable of the Deuteron in its ground state.

## Outline of isotopic methods

In this section, we outline the *isomathematical and isomechanical branch of hadronic mechanics*^14^ (see^18–20^ for a recent review). Let $\xi : \{(a, b, \ldots ), \times , I\}$ be the universal enveloping associative algebra of quantum mechanics with generic elements *a*, *b* product $ab = a\times b$ and conventional multiplicative unit $I = 1$. The central assumption of isotopic methods, first introduced in p. 71, Vol. II^13^, is the EPR completion of $\xi : \{(a, b, ..), \times , I\}$ into the isoassociative algebra ${\hat{\xi }}:\{(a, b\ldots ), \star , {\hat{I}}\}$ characterized by the axiom-preserving product called *isoproduct*

5 $$\begin{aligned} a \star b = a {\hat{T}} b, \quad{\hat{T}} > 0, \end{aligned}$$

where the quantity ${\hat{T}}$, called the *isotopic element,* is positive-definite but otherwise possesses an arbitrary functional dependence on local variables such as relative coordinates *r*, momenta *p*, wavefunctions $\psi$, etc. hereon tacitly implied.

Completion (5) of the product evidently implies the corresponding compatible completion of the multiplicative unit 1 into the *isounit* of ${\hat{\xi }}: \{(a, b,\ldots ), \star , {\hat{I}}\}$^37^

6 $$\begin{aligned} {\hat{I}} = 1/{\hat{T}} > 0, \quad {\hat{I}} \star a = a \star {\hat{I}} = a\, \forall \, a \in {\hat{\xi }}, \end{aligned}$$

with realizations of the type^15^

7 $$\begin{aligned} {\hat{T}} = 1/{\hat{I}} = \Pi _{\rho = 1, 2,\ldots ,N} Diag. \left( {1\over n_{1\rho }^2}, {1\over n_{2\rho }^2}, {1\over n_{3\rho }^2}, {1\over n_{4,\rho }^2}\right) e^{-\Gamma } > 0, \end{aligned}$$

where: $n_{k\rho }^2, k = 1, 2, 3$ represents the nucleon semi-axes which are hereon assumed to be the same for all nucleons, with normalization for the sphere

8 $$\begin{aligned} n_1^2 + n_2^2 + n_3^2 = 3, \end{aligned}$$

where $n_3^2$ is directed along the spin axis; $n_{4\rho }^2$ represents the nucleon density also assumed for simplicity to be the same for all nucleons; and $\Gamma$ represents non-linear, non-local and non- potential interactions.

Consequently, the representation of extended particles via isotopic methods requires *two operators,* the conventional Hamiltonian *H* for the representation of Hamiltonian interactions, and the isotopic element ${\hat{T}}$ for the representation of the dimension, shape and density of particles as well as their non-Hamiltonian interactions.

Via the use of isomathematics and isomechanics, Santilli achieved in 1994^38^ (see also Ref.^39^ and the review/update^20^) an exact representation of the magnetic moment of the Deuteron and of other stable nuclei via a quantitative representation of the Fermi–Weisskopf hypothesis^4,3^ permitted by realizations (7).

Since, by main assumption, the spin of nucleons remains $S = 1/2$, and the magnetic moment of nucleons is linked to the spin via the known gyromagnetic factor *g*

9 $$\begin{aligned} \mu = g S, \end{aligned}$$

Santilli^38^ reached the following completion of the gyromagnetic factor for individual nucleons (called *iso-renormalization* because it is characterized by non-Hamiltonian interactions)

10 $$\begin{aligned} \mu = {n_3\over n_4} g S. \end{aligned}$$

The knowledge of the nucleon density $n_4$, from the fit of particle physics experiments^40,41^ and of deviation (4) for the deformation of $n_3$, allowed a numeric representation of the magnetic moment of the Deuteron and of other nuclei.

**Figure 2:**
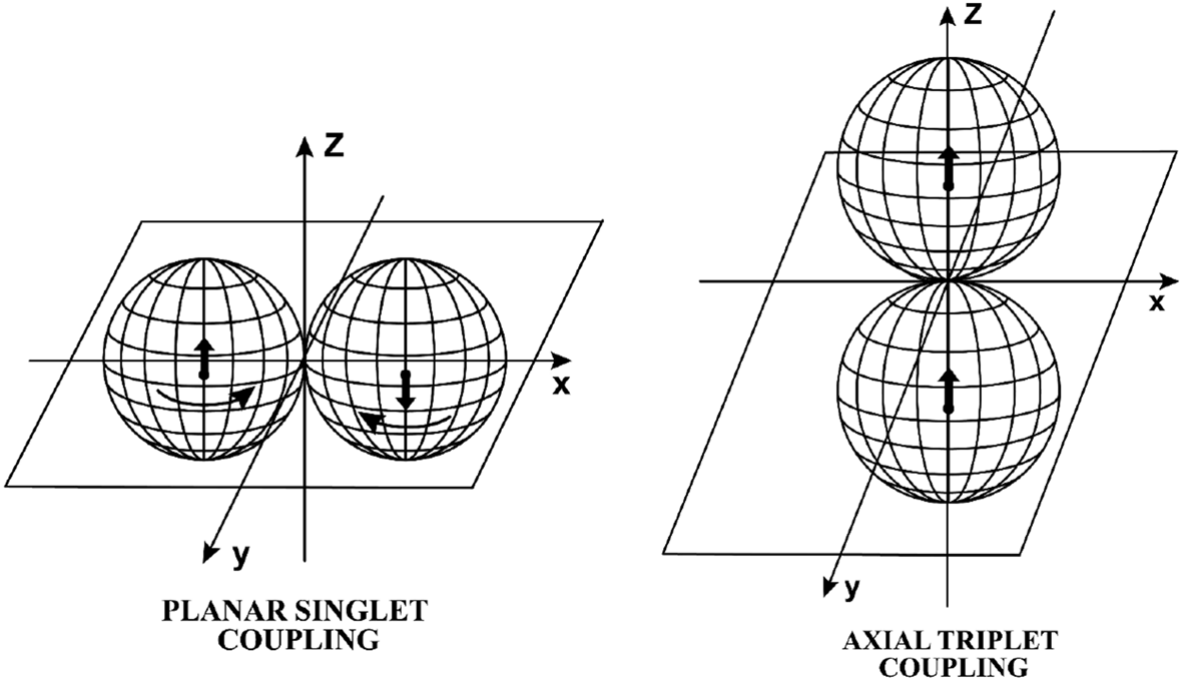
In this figure, we illustrate the sole stable couplings predicted by hadronic mechanics for nuclear fusions (see Fig. 3, p. 152, of Ref.^42^), which include: the conventional singlet coupling with a plane symmetry and antiparallel spins (on the left) called ‘planar singlet coupling’; and the novel triplet coupling with a symmetry axis and parallel spins (on the right) called ‘axial triplet coupling’. The remaining possible couplings (which are given by the planar triplet with parallel spins and the axial singlet with antiparallel spins not shown in this figure) have been proved to be unstable, and therefore are ignored for nuclear fusions^42^.

**Figure 3:**
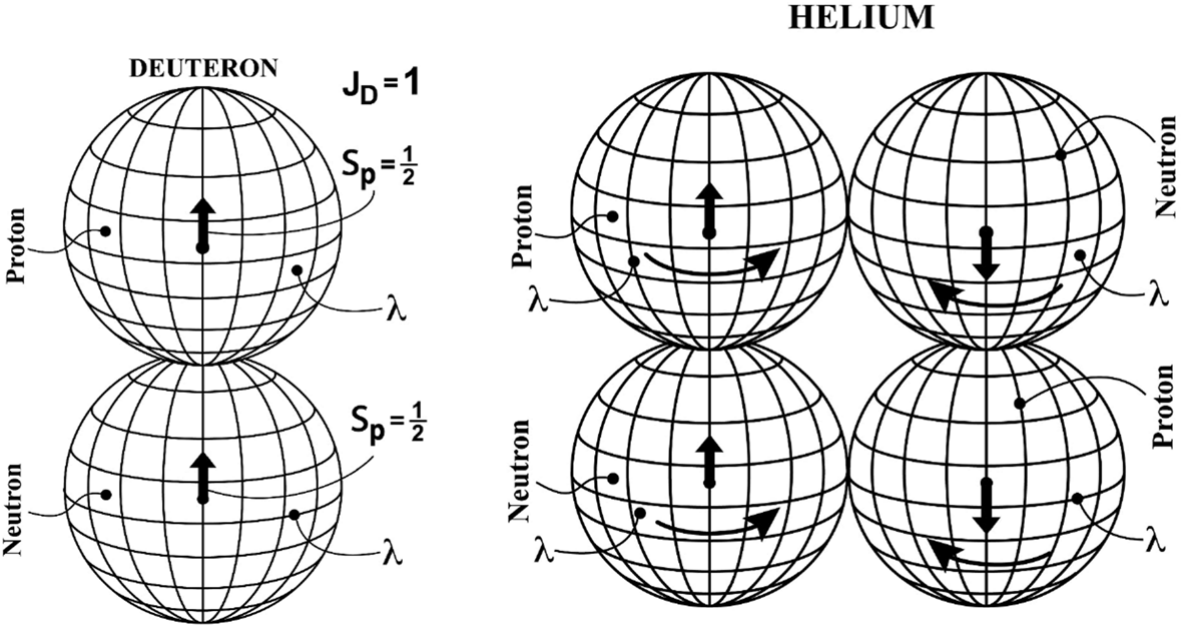
In this figure, we illustrate on the left the structure model of the Deuteron permitted by hadronic mechanics as a bound state under strong interactions of a proton and a neutron in ‘axial triplet coupling’ (Figure 2). This model has permitted the first known representation of the spin $S_d = 1$ of the Deuteron in its true ground state (Section 8.2.5, p. 90 on of Refs.^15,31^), here denoted $2-H-1_\lambda$ to indicate the presence of Bohm’s hidden variable in the spin of particles according to the iso-Pauli matrices of Eq. (18)^16^. In the right, we illustrate the structure model of the Helium, here denoted $4-He-2_\lambda$, as a bound state under strong interactions of two nuclei $2-H-1_\lambda$ with antiparallel spins. This model allows a representation of the null spin and magnetic moment of Helium in its ground state.

The representation of the spin $S_d = 1$ of the Deuteron as a stable two-body problem of spin 1/2 particles *in the ground state* (i.e., with null value of the angular momentum) was first reached in Section 8.2.5, p. 90 on of Ref.^15^, and then studied in detail in Ref.^31^ jointly with the first known achievement of the numeric value of Bohm’s hidden variable $\lambda$ for the Deuteron, thanks to the *axial triplet coupling* permitted by hadronic mechanics with the proton and the neutron coupled along their symmetry axis with parallel spin^42^ (Figs. 2 and 3).

Isotopic methods also allowed a representation of the spin $S_d = 1$ of the Deuteron in its ground state thanks to the axial triplet coupling of the proton and the neutron^42^ (see Section 2.7.2 of Ref.^20^, Section 8.2.5 of Ref.^15^ and the latest Ref.^31^).

(iii) Accurate fits without the conjecture of unknown parameters or particles of the Bose-Einstein correlation^40,41^, the behavior of the mean lives of unstable hadrons with speed^43,44^, the anomalous magnetic moment of the muons^45^, and new vistas for the possible achievement of controlled nuclear fusions^15,42^.

Subsequently, Santilli^16^ achieved in 1998 a verification of the EPR argument via the Lie-isotopic completion of the Lie algebras of quantum mechanics (see Refs.^13,14^, independent study^24^ and the recent update^46^) applied to the isotopies $\hat{SU}(2)$ of the *SU*(2)-spin algebra (see Section 3 of Ref.^19^ for a recent review). The main results of this study are:

(I) The confirmation of the validity of Bell’s inequality^47^ for point-like particles under electromagnetic interactions and its *inapplicability* (rather than its violation) for extended particles under strong interactions with Hamiltonian and non-Hamiltonian interactions and consequential recovering of the classical picture for systems of extended spin 1/2 particles under strong interactions^16^.
(II) The progressive recovering of Einstein’s determinism^2^ with the increase of the density of strongly interacting systems, and its full recovering at the limit of Schwartzschild horizon^17^.
(III) The conversion of quantum mechanical strongly divergent perturbative series into strongly convergent isotopic series, evidently in view of the very small value of the isotopic element ${\hat{T}}$ of Eq. (9), sandwiched in between all products. Apparently, this result sets up the foundations for the elimination of divergencies in particle physics (see Chapters 11 and 12, Vol. II of Ref.^14^ and upgrade^15^). As an example isotopic methods achieved the first known *attractive force* between the *identical electrons* of valence bonds (see Chapter 4 on of^48^) whose resulting *strong valance bond* permitted the first known exact representation of experimental data for the hydrogen^49^ and water^50^ molecules without divergent perturbative series.

## Representation of nuclear magnetic moments and spins via geometric Clifford algebras

Consider the fundamental representation of the *SU*(2)-spin algebra on $\mathbb {R}^3$ given by the celebrated *Pauli’s matrices* $\sigma _k$, here recalled with the symbols of Clifford’s geometric algebra^32–36^

11 $$\begin{aligned} e_1= \left( \begin{array}{cc} 0&{} 1\\ 1 &{} 0 \end{array}\right) ,\quad e_2 = \left( \begin{array}{cc} 0&{} -i\\ i &{} 0 \end{array} \right) ,\quad e_3 = \left( \begin{array}{cc} 1 &{} 0\\ 0&{} - 1 \end{array}\right) , \end{aligned}$$

(where $e_3$ refers hereon to the spin direction) with commutation rules

12 $$\begin{aligned}{}[e_i , e_j] =e_i e_j - e_j e_i = i 2 \epsilon _{ijk} e_k, \end{aligned}$$

and enveloping associative algebra $\xi$ with products of the type $e_i e_j$. The spin $S = 1/2$ can be characterized by the eigenvalue equations on a Hilbert space $\mathbb {H}$ with basis $| b>$

13 $$\begin{aligned} \begin{array}{l} S_k= {1\over 2 } e_k,\\ e_3 | b> =\pm | b> , \\ e^{{\hat{2}}} | b> = (e_1 e_1 + e_2 e_2 + e_3 e_3) | b> = 3 |b>. \end{array} \end{aligned}$$

Santilli introduced in the 1983 Vol. II of^13^ the EPR completion ${\hat{\xi }}$ of the universal enveloping algebra $\xi$ of Lie algebras, including that of *SU*(2) via *non-unitary transformations*

14 $$\begin{aligned} U U^\dagger \ne I, \end{aligned}$$

of the associative product, resulting in the iso-product of ${\hat{\xi }}$

15 $$\begin{aligned} U ( e_i e_j) U^\dagger&= {} (U e_i U^\dagger ) (U U^\dagger )^{-1} (U e_j U^\dagger ) = {\hat{e}}_i {\hat{T}} {\hat{e}}_j = {\hat{e}}_i \star {\hat{e}}_j, \\ {\hat{e}}_i&= U e_i U^\dagger ,\quad {\hat{e}}_j = U e_j U^\dagger ,\quad {\hat{T}} = (U U^\dagger )^{-1} \end{aligned}$$

with ensuing non-unitary transformation of the multiplicative unit *I* of *SU*(2) into the iso-unit of $\hat{SU}(2)$

16 $$\begin{aligned} U I U^\dagger = {\hat{I}} = 1/{\hat{T}}. \end{aligned}$$

Santilli then introduced in the 1995 Vol. II of^14^ the realization of the isotopic element ${\hat{T}}$ and isounit ${\hat{I}} = 1/{\hat{T}}$ in terms of Bohm’s hidden variables $\lambda$^11^

17 $$\begin{aligned} {\hat{I}} = Diag. (\lambda ^{-1}, \lambda ), \quad {\hat{T}} = Diag. (\lambda , \lambda ^{-1}), \quad Det {\hat{I}} = 1, \end{aligned}$$

resulting in the following *iso-Pauli matrices* first introduced in Eq. (6.8.20), p. 254, Vol. II^14,16^

18 $$\begin{aligned} {\hat{e}}_1= \left( \begin{array}{cc} 0&{} \lambda \\ \lambda ^{-1}&{} 0 \end{array}\right) ,\quad {\hat{e}}_2 = \left( \begin{array}{cc} 0&{} -i \lambda \\ i \lambda ^{-1}&{} 0 \end{array} \right) ,\quad {\hat{e}}_3 = \left( \begin{array}{cc} \lambda ^{-1}&{} 0\\ 0&{} -\lambda \end{array}\right) . \end{aligned}$$

The classification of all possible isotopies of Pauli’s matrices was subsequently provided in Section 3, Ref.^19^.

It should be noted that Santilli derived iso-Pauli matrices (18) from the isotopy in $(3+1)$-dimensions of Dirac’s gamma matrices thanks to the isotopy of the spinorial covering of the Poincaré symmetry $\mathcal{{\hat{P}}}(3.1)$ Ref.^52^.

Consequently, iso-Pauli matrices (18) are invariant over time when formulated on the Hilbert-Myung-Santilli isospace $\mathcal{{\hat{H}}}$^53^ over the isofield of isocomplex isonumbers $\mathcal{{\hat{C}}}$^37^.

Note also the isomorphism between the Lie-Santilli isoalgebra $\hat{SU}(2)$ and the conventiional Lie algebra *SU*(2) which is established by the preservation under isotopy of the *SU*(2) structure constants in the iso-commutation rules

19 $$\begin{aligned}{}[{\hat{e}}_i , {\hat{e}}_j]^* = {\hat{e}}_i \star {\hat{e}}_j - {\hat{e}}_j \star {\hat{e}}_i = {\hat{e}}_i {\hat{T}} {\hat{e}}_j - {\hat{e}}_j {\hat{T}} {\hat{e}}_i = i 2 \epsilon _{ijk} {\hat{e}}_k. \end{aligned}$$

It is important to note that, despite their seemingly generalized structure (18), the iso-Pauli matrices do indeed characterize the spin $S = 1/2$ of nucleons when formulated via isomathematics^14,51^ on isospace $\mathcal{{\hat{H}}}$ with isobasis $| {\hat{b}}>$

20 $$\begin{aligned} \begin{array}{l} \hat {S} _k= {1\over 2 }{\hat{e}}_k,\\ \hat{e}_3 {\hat{T}} |{\hat{b}}> =\pm |{\hat{b}}> , \\ {\hat{e}}^{{\hat{2}}} = ({\hat{e}}_1 {\hat{T}} {\hat{e}}_1 + {\hat{e}}_2 {\hat{T}} {\hat{e}}_2 + {\hat{e}}_3 {\hat{T}} {\hat{e}}_3) {\hat{T}} |{\hat{b}}> = 3 |{\hat{b}}>. \end{array} \end{aligned}$$

The primary meaning of Santilli’s iso-Pauli matrices (18) is therefore that of introducing the new notion of *hadronic spin* whose numeric values are conventional yet possessing a novel degree of freedom given by the explicit and concrete realization of Bohm hidden variable $\lambda$.

Independently from the above studies, Sobczyk^32–36^ studied the representations of the conventional Pauli matrices (11) in terms of Clifford’s algebra $\mathbb {G}_3=\mathbb {G}_3(\mathbb {R}^3 )$ with standard basis

21 $$\begin{aligned} \mathbb {G}_3 = \mathbb {G}_3(\mathbb {R}^3 ):=span_\mathbb {R}\{ 1,\textbf{e}_1,\textbf{e}_2, \textbf{e}_3, \textbf{e}_1\textbf{e}_2,\textbf{e}_1\textbf{e}_3, \textbf{e}_2 \textbf{e}_3, i:=\textbf{e}_1\textbf{e}_2 \textbf{e}_3 \}, \end{aligned}$$

where $\textbf{e}_1, \textbf{e}_2,\textbf{e}_3$ are interpreted as *unit vectors* along the *x*, *y*, *z*-axes of the coordinates space of te Euclidean 3-space $\mathbb {R}^3$, with properties

22 $$\begin{aligned} \textbf{e}_1^2 = \textbf{e}_2^2 = \textbf{e}_3^2 = 1. \end{aligned}$$

We then interpret the quantities

23 $$\begin{aligned} \textbf{e}_{12}=\textbf{e}_1\textbf{e}_2=-\textbf{e}_{21},\quad \textbf{e}_{13}=\textbf{e}_1 \textbf{e}_3 ,\quad \textbf{e}_{23}=\textbf{e}_2\textbf{e}_3, \end{aligned}$$

to be *unit bivectors* defining the oriented *planar directions* of the *xy*-, *xz*-, and *yz*-planes, respectively, with squares

24 $$\begin{aligned} \textbf{e}_{12}^2 = \textbf{e}_1 \textbf{e}_2 \textbf{e}_1 \textbf{e}_2=-\textbf{e}_1 \textbf{e}_2 \textbf{e}_2 \textbf{e}_1 =-\textbf{e}_1^2\textbf{e}_2^2 =-1. \end{aligned}$$

The *unit trivector* $i=\textbf{e}_{123}=\textbf{e}_1\textbf{e}_2\textbf{e}_3$, in the center of the algebra $\mathbb {G}_3$, also has square $-1$.

The geometric algebra $\mathbb {G}_3$ is isomorphic to the familiar $2\times 2$ complex matrix algebra ${{{\mathcal {M}}}}_2(\mathbb {C})$, which defines the *coordinate matrices* of $\mathbb {G}_3$ with respect to the *spectral basis*,

25 $$\begin{aligned} \left( \begin{array}{cc} u_+ &{} \textbf{e}_1 u_- \\ \textbf{e}_1 u_+ &{} u_- \end{array}\right) , \end{aligned}$$

where $u_{\pm }:=\frac{1}{2}(1\pm \textbf{e}_3 )$ are *mutually annihilating idempotents* satisfying the rules

26 $$\begin{aligned} u_+u_-=0,\quad u_+ +u_-=1, \quad \textbf{e}_3 u_\pm =\pm u_\pm , \end{aligned}$$

as can be easily verified.

The complex $2\times 2$ matrix

27 $$\begin{aligned}{}[A]=\left( \begin{array}{cc} a_{11} &{} a_{12} \\ a_{21} &{} a_{22} \end{array}\right) , \end{aligned}$$

where $i\equiv \textbf{e}_{123}$, is the *coordinate matrix* of the geometric number $A \in \mathbb {G}_3$ with explicit form,

28 $$\begin{aligned} A = a_{11}u_+ + a_{12}\textbf{e}_1 u_- +a_{21}\textbf{e}_1 u_++ a_{22}u_-. \end{aligned}$$

According to the above realization of our Clifford geometric algebra, Pauli matrices (11) are the coordinate matrices of the unit vectors $\textbf{e}_1,\textbf{e}_2,\textbf{e}_3$ defined by

29 $$\begin{aligned} \textbf{e}_1=\textbf{e}_1 u_+ + \textbf{e}_1 u_-, \quad \textbf{e}_2=i \textbf{e}_1 u_+ - i\textbf{e}_1 u_-,\quad \textbf{e}_3 = u_+-u_-, \end{aligned}$$

where again $i = \textbf{e}_1 \textbf{e}_2 \textbf{e}_3$ is the unit trivector in the geometric algebra $\mathbb {G}_3$. Thus, the so called *Pauli vectors* $e_k\equiv \textbf{e}_k, k=1,2,3$, are identified with the unit vectors $\textbf{e}_k$ along the *xyz*-axes (for details, see Refs.^32–36^.

By upgrading and extending Ref.^31^, we now study the representation of nuclear magnetic moments via the reformulation of iso-Pauli matrices (18)^16^ in terms of geometric algebra (21)^33^. For this purpose, we note that when formulated on the *associative envelope* $\xi$, the iso-Pauli matrices satisfy all algebraic properties of the conventional Pauli matrices. Consequently, we here introduce the *representation of iso-Pauli matrices* (18) *in terms of Clifford geometric algebra* $\tilde{\mathbb {G}}_3 = \tilde{\mathbb {G}}_3(\mathbb {R}^3 )$ with the iso-basis

30 $$\begin{aligned} \tilde{\mathbb {G}}_3 = \tilde{\mathbb {G}}_3(\mathbb {R}^3 ):=span_\mathbb {R}\{ 1, {\hat{\textbf{e}}}_1, {\hat{\textbf{e}}}_2, {\hat{\textbf{e}}}_3, {\hat{\textbf{e}}}_1 {\hat{\textbf{e}}}_2,{\hat{\textbf{e}}}_1{\hat{\textbf{e}}}_3, {\hat{\textbf{e}}}_2 {\hat{\textbf{e}}}_3, i: = {\hat{\textbf{e}}}_1 {\hat{\textbf{e}}} _2 {\hat{\textbf{e}}}_3 \}, \end{aligned}$$

and main properties equivalent to (22)–(29), including their interpretation in terms of vectors, bivectors and trivectors,

31 $$\begin{aligned} {\hat{\textbf{e}}}_1^2= & {} {\hat{\textbf{e}}}_2^2 = {\hat{\textbf{e}}}_3^2 = 1, \end{aligned}$$

32 $$\begin{aligned} {\hat{\textbf{e}}}_{12}= & {} {\hat{\textbf{e}}}_1 {\hat{\textbf{e}}}_2 = - {\hat{\textbf{e}}}_{21},\quad {\hat{\textbf{e}}}_{13} = {\hat{\textbf{e}}}_1 {\hat{\textbf{e}}}_3 ,\quad {\hat{\textbf{e}}}_{23} = {\hat{\textbf{e}}}_2 {\hat{\textbf{e}}}_3, \end{aligned}$$

33 $$\begin{aligned} {\hat{\textbf{e}}}_{12}^2= & {} {\hat{\textbf{e}}}_1 {\hat{\textbf{e}}}_2 {\hat{\textbf{e}}}_1{\hat{\textbf{e}}}_2= - {\hat{\textbf{e}}}_1 {\hat{\textbf{e}}}_2 {\hat{\textbf{e}}}_2 {\hat{\textbf{e}}}_1 = - {\hat{\textbf{e}}}_1^2 {\hat{\textbf{e}}}_2^2 = - 1. \end{aligned}$$

The *standard basis* of unit iso-vectors $\{{\hat{\textbf{e}}}_1, {\hat{\textbf{e}}}_2, {\hat{\textbf{e}}}_3\}$ define the the *x*, *y*, *z* iso-coordinate axes, respectively. The *iso-spectral basis* is

34 $$\begin{aligned} \left( \begin{array}{cc} {\hat{u}}_+ &{} {\hat{\textbf{e}}}_{1} {\hat{u}}_- \\ {\hat{\textbf{e}}}_1 {\hat{u}}_+ &{} {\hat{u}}_- \end{array}\right) , \end{aligned}$$

where ${\hat{u}}_{\pm }:=\frac{1}{2}(1\pm {\hat{\textbf{e}}}_3 )$ are *mutually annihilating iso-idempotents*. In the standard iso-basis of ${\hat{\mathbb {G}}}_3$,

35 $$\begin{aligned} \{{\hat{\textbf{e}}}_1, \ {\hat{\textbf{e}}}_2=i {\hat{\textbf{e}}}_1{\hat{\textbf{e}}}_3 ,\ {\hat{\textbf{e}}}_3 \}, \end{aligned}$$

where

36 $$\begin{aligned} {\hat{i}} = {\hat{\textbf{e}}}_1 {\hat{\textbf{e}}}_2 {\hat{\textbf{e}}}_3, \end{aligned}$$

is the *unit iso-trivector* of the associative geometric algebra $\tilde{\mathbb {G}}_3$. It must be remembered that for the iso-basis vectors ${\hat{\textbf{e}_k}}$,

37 $$\begin{aligned} {\hat{\textbf{e}}}_k^2 = {\hat{\textbf{e}_k}} \star {\hat{\textbf{e}}}_k = 1, \end{aligned}$$

for $k=1,2,3$, where the $\star$ denotes the iso-geometric product. The products in Eqs. (35) and (36) are also iso-products.

We now show that the hidden variable $\lambda$ of Ref.^16^ can provide a second representation of the deformation of the magnetic moment of nucleons of Refs.^38,39^ with consequential exact representation of nuclear magnetic moments^31^.

By introducing the realization of the hidden variable $\lambda$

38 $$\begin{aligned} \lambda = e^\phi \ge 0, \end{aligned}$$

with respect to the standard basis (21), the *iso-Pauli unit* ${\hat{I}}$, the *iso-reciprocal* ${\hat{T}}$, and the *iso-vector basis* $\{ {\hat{\textbf{e}}}_k\}$, expressed in the geometric algebra $\mathbb {G}_3$, are given by

39 $$\begin{aligned} {\hat{I}}=\cosh \phi + \textbf{e}_3 \sinh \phi = e^{\phi \textbf{e}_3 }, \quad {\hat{T}}=\cosh \phi - \textbf{e}_3 \sinh \phi = e^{-\phi \textbf{e}_3 }, \end{aligned}$$

which are a different expression of, but equivalent to, Eqs. (16) and (17). Consequently

40 $$\begin{aligned} {\hat{\textbf{e}}}_1 = \textbf{e}_1 {\hat{I}} ={\hat{T}} \textbf{e}_1 , \quad \ {\hat{\textbf{e}}}_2 := \textbf{e}_2 {\hat{I}} = {\hat{T}} \textbf{e}_2, \quad {\hat{\textbf{e}}}_3 := \textbf{e}_3 {\hat{I}} = {\hat{I}} \textbf{e}_3, \end{aligned}$$

which expression is equivalent to the second part of Eq. (15).

By recalling that $e_3$ characterizes the nucleon spin $S = 1/2$, we reach the important result that the *replacement of the standard basis of the geometric Clifford algebra* $\mathbb {G}_3$ *of Pauli’s matrices* (11) *with their iso-Pauli form* (18), *implies the EPR completion of* $e_3$ *into the expression defined on the iso-basis* $| {\hat{b}}>$ *of the iso-Pauli matrices*

41 $$\begin{aligned} {\hat{\textbf{e}}}_3 | {\hat{b}}> = e_3 {\hat{I}} | {\hat{b}}> = e_3 e^{\phi e_3 } | {\hat{b}}>. \end{aligned}$$

Recall that the quantum mechanical (qm) relationship between magnetic moments $\mu$ and spins *S* occurs via the gyromagnetic factor *g* of Eq. (9), and that the corresponding relation for the isotopic branch of hadronic mechanics (hm) is given by an expression of the type^38^

42 $$\begin{aligned} \mu _{hm} | {\hat{b}}> = {\hat{S}} | {\hat{b}}> = K g S | {\hat{b}}>, \end{aligned}$$

where *K* is an isorenormalization constant of the gyronamgentic factor *g* created by the the new notion of spin 1/2 represented by iso-Pauli matrices (18) with Bohm’s hidden variable $\lambda$^14^.

By using property (41), we reach the relation

43 $$\begin{aligned} \mu _{hm} | {\hat{b}}> = e^{\phi e_3 } \mu _{qm} | {\hat{b}}> = e^{\phi e_3 } g S | {\hat{b}}>. \end{aligned}$$

Recall also that: 1) Bohm’s hidden variable $\lambda$ is associated with the *spin* of a particle according to Eq. (18); 2) The proton and the neutron have the same spin 1/2 and essentially the same mass, thus being characterized by the same $\lambda$; and 3) The quantum mechanical representation of the magnetic moment of the Deuteron is *in excess* of about $3\%$ according to Eq. (4). By also selecting the value for conformity with the selected spin orientation (Fig. 3)

44 $$\begin{aligned} e_3 | {\hat{b}}> = - | {\hat{b}}>, \end{aligned}$$

we can write the expression per each nucleon

45 $$\begin{aligned} \mu _{hm, k} \approx (1 + \phi e_3 ) \mu _{qm, k} = ( 1 - \phi ) \mu _{qm. k} , \quad k = p, n, \end{aligned}$$

from which we obtain the isorenormalized values of the magnetic moment of the proton and of the neutron

46 $$\begin{aligned} {\hat{\mu }}_p = + (1 - \phi ) 2.79285 \,\mu _N ,\quad {\hat{\mu }}_n = - (1 - \phi )\, 1.91304 \,\mu _N , \end{aligned}$$

with corresponding value for the magnetic moment of the deuteron

47 $$\begin{aligned} \mu _D^{hm}&= (1 - \phi )~ 2.79285 - (1 - \phi ) ~1.91304 ~\mu _N \\ &= {} (1 - \phi ) ~0.87981 ~ \mu _N = \mu _D^{ex} = 0.85647 ~ \mu _N, \end{aligned}$$

From this, we obtain the numeric value

48 $$\begin{aligned} \phi = 1 - 0.87981 / 0.85647 = 1 - 0.02334 = 0.97666, \end{aligned}$$

with consequential confirmation of the numeric value of Bohm’s hidden variable for the Deuteron first achieved in Ref.^31^

49 $$\begin{aligned} \lambda = e^{\phi } = e^{0.97666} = 2.65557. \end{aligned}$$

Its invariance over tine follows from the derivation of iso-Pauli matrices (18) from the Lie-Santilli isosymmetry $\mathcal{{\hat{P}}}(4.1)$^16,52^.

The representation of the magnetic moment of $4-He-2$ is a consequence (Fig. 3), but that for other stable nuclei requires the still missing consistent representation of nuclear spins which was initiated in Ref.^54^ and will be studied in a forthcoming paper.

We should finally note that we *have not* used the full isotopic Clifford algebra $\hat{\mathbb {G}}$ introduced by da Rocha and Vaz Jr. (see^55^ with important applications to flavor quark theories), since we have merely introduced the *conventional* geometric formulation $\tilde{\mathbb {G}}_3$ in terms of iso-Pauli matrices (18). This is due to the fact that the full geometric isotopy $\hat{\mathbb {G}}_3$ of $\mathbb {G}_3$ would have required the use of isoproduct (5) with the isotopic element ${\hat{T}} = e^{-\phi e_3} = 1/{\hat{I}}$, and the consequential lack of representation in Eq. (41) of the magnetic moment of the Deuteron (3) for spin $S_d = 1$ in the ground state.

The understanding is however that the full iso-Clifford isogeometric isoalgebra $\hat{\mathbb {G}}_{3N}$ is expected to be important for the numerically exact and time invariant representation of the spins and magnetic moments of nuclei with $N \ge 2$ nucleons.

## Concluding remarks

Various experiments have established that the quantum mechanical prediction of the magnetic moment of the Deuteron is about $3\%$ in *excess* of the experimental value, Eq. (4), with bigger deviations for bigger nuclei (Fig. 1)^1^.

The basic event studied in this paper to achieve an exact representation of nuclear magnetic moments consists of the fact that a rotating charge distribution creates a magnetic field along the rotation axis. When the charge distribution is deformed into a prolate ellipsoid, the magnetic field decreases, and when it is deformed into an oblate ellipsoid it increases (Fig. 2).

This evidence led Fermi^4^, Weisskopf^3^ and other founders of nuclear physics, to suggest the hypothesis that the difference between quantum mechanical predictions of nuclear magnetic moments and their experimental values may be due to deformations of the charge distributions of protons and neutrons under strong nuclear interactions.

The study of this historical hypothesis required the completion of quantum mechanics according to the 1935 historical argument by Einstein, Podolsky and Rosen, that *quantum mechanics is not a complete theory*^2^, because quantum mechanics can only characterize *point-like particles* that, as such, cannot be deformed.

Extensive studies over the past half a century conducted by R. M. Santilli and other scholars^12–22^ (see Refs.^23–30^ for independent reviews) have shown that the *extended* character of nucleons, their *deformations* and their consequential *non-Hamiltonian interactions* can be represented via the *axiom-preserving, non-unitary completion* of the universal enveloping associative algebra of quantum mechanics in terms of associative isoproduct $a {\hat{T}} b$ of Eq. (5) where ${\hat{T}}$, called the *isotopic element,* is positive-definite, yet possesses an arbitrary functional dependence on any needed local variable.

The dimension, shape and density of nucleons as well as their deformations and non-Hamiltonian interactions are then represented with the new isotopic element ${\hat{T}}$ according to realizations of type (7).

These studies lead to the construction of the axiom-preserving non-unitary completion of quantum mechanics into *hadronic mechanics*^13–15^ (see Refs.^18–20^ for a recent review) which coincides with quantum mechanics at the abstract, realization-free level. Via the use of the isomathematical and isomechanical branch of hadronic mechanics, Santilli^38^ achieved in 1994 the first known numerically exact and time invariant representation of the magnetic moment of the Deuteron and other nuclei according to the Fermi- Weisskopf hypothesis via a mere 1.5% prolate deformation of the charge distribution of protons and neutrons when they are members of a nuclear structure.

In subsequent studies, Santilli^16^ introduced the broader notion of *hadronic spin* for extended particles under strong interactions, which spin is illustrated by *iso-Pauli matrices* (18), possess conventional spin values, and exhibits an explicit and concrete realization of Bohm’s hidden variable $\lambda$^11^ in term of the isotopic element ${\hat{T}}$, thus being hidden in the axiom of associativity.

In this paper we have shown, apparently for the first time, that the indicated notion of hadronic spin with Bohm’s hidden variable $\lambda$ allows a second numerically exact and time invariant representation of the magnetic moment of the Deuteron and of other nuclei. The representation has been possible thanks to the reformulation of Santilli’s isotopy of Pauli matrices (first introduced in p. 254, Vol. II of Ref.^14^ and used in Ref.^16^ for the verification of the EPR argument) in terms of the conventional Clifford’s algebras, Refs.^32–36^.

In a nutshell, we can say that the Copenhagen interpretation of quantum mechanics deals with the simplest possible realization of quantum axioms, while the EPR completion of quantum into hadronic mechanics deals with progressively broader realizations of the same axioms for systems with progressively increasing complexity.

## Data Availability

The datasets used and/or analysed during the current study are available from the corresponding author on reasonable request.
